# Role of Ginseng, Quercetin, and Tea in Enhancing Chemotherapeutic Efficacy of Colorectal Cancer

**DOI:** 10.3389/fmed.2022.939424

**Published:** 2022-06-20

**Authors:** Linxian Zhao, Hongyu Zhao, Yongqing Zhao, Mingxiu Sui, Jinping Liu, Pingya Li, Ning Liu, Kai Zhang

**Affiliations:** ^1^Department of General Surgery, The Second Hospital of Jilin University, Changchun, China; ^2^Gastroenterology and Center of Digestive Endoscopy, The Second Hospital of Jilin University, Changchun, China; ^3^Research Center of Natural Drugs, School of Pharmaceutical Sciences, Jilin University, Changchun, China; ^4^Department of Central Laboratory, The Second Hospital of Jilin University, Changchun, China

**Keywords:** colorectal cancer, ginseng, quercetin, tea, chemotherapy, chemoresistance

## Abstract

As the most common gastrointestinal malignancy, colorectal cancer (CRC) remains a leading cause of cancer death worldwide. Although multimodal chemotherapy has effectively improved the prognosis of patients with CRC in recent years, severe chemotherapy-associated side effects and chemoresistance still greatly impair efficacy and limit its clinical application. In response to these challenges, an increasing number of traditional Chinese medicines have been used as synergistic agents for CRC administration. In particular, ginseng, quercetin, and tea, three common dietary supplements, have been shown to possess the potent capacity of enhancing the sensitivity of various chemotherapy drugs and reducing their side effects. Ginseng, also named “the king of herbs”, contains a great variety of anti-cancer compounds, among which ginsenosides are the most abundant and major research objects of various anti-tumor studies. Quercetin is a flavonoid and has been detected in multiple common foods, which possesses a wide range of pharmacological properties, especially with stronger anti-cancer and anti-inflammatory effects. As one of the most consumed beverages, tea has become particularly prevalent in both West and East in recent years. Tea and its major extracts, such as catechins and various constituents, were capable of significantly improving life quality and exerting anti-cancer effects both in *vivo* and in *vitro*. In this review, we mainly focused on the adjunctive effects of the three herbs and their constituents on the chemotherapy process of CRC.

## Introduction

Colorectal cancer (CRC) is the third most common cancer globally and one of the leading causes of health burden on society (1). The latest epidemiological data show that the incidence of CRC is rapidly increasing year by year, and the number of young patients aged 20–40 years old has increased quickly (2). For early-stage CRC, surgical resection of the primary tumor is the main treatment method, and adjuvant chemotherapy can prolong the survival times of patients (3, 4). In terms of advanced CRC or metastatic CRC, the survival rate is less than 10%, and the primary treatment strategies include radiotherapy and chemotherapy. CRC represents a heterogeneous disease with distinct disease mechanisms and prognoses.

It has been confirmed that multiple factors were involved in CRC development and progression, such as genetic alterations, gut microbiota, chronic inflammation, environmental influence, and others (1, 3). However, the exact mechanisms underlying the onset of colorectal cancer are still unknown. With the development of precision medicine and personalized medicine, chemotherapy plays an increasingly important role in CRC administration. Especially for advanced patients, chemotherapy offers the only possibility of a cure. However, the clinical application of chemotherapeutic regimens is mainly limited by their side effects and toxicity. Therefore, urgent research is needed to discover more adjuvant chemotherapy compounds to enhance the tumoricidal effects at low doses (5).

**Figure 1:**
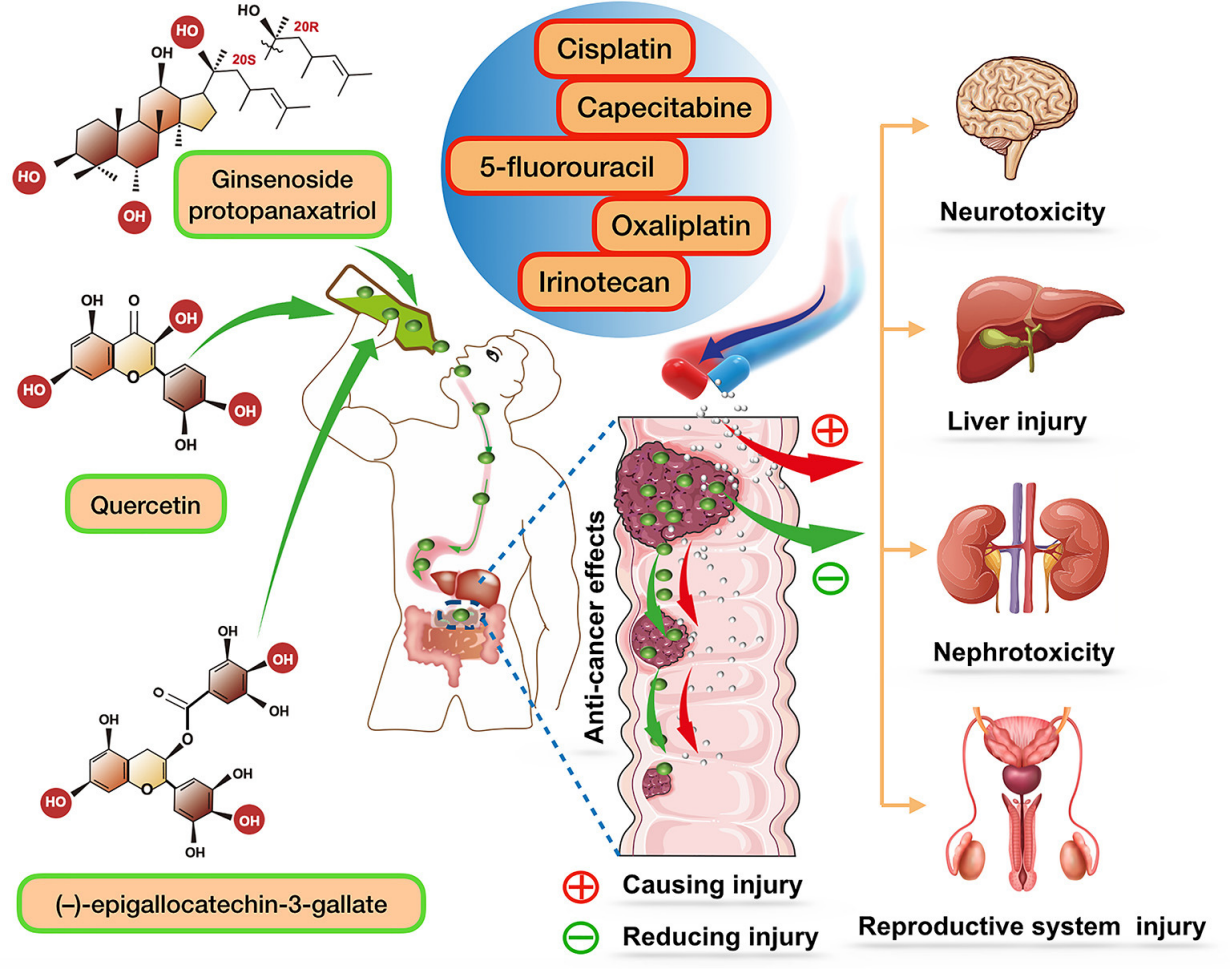
The role of ginsenoside protopanaxatriol (one major extract of ginseng), quercetin, and (–)-epigallocatechin-3-gallate (one major extract of tea) in enhancing chemotherapeutic efficacy of various chemotherapy drugs, and together reducing their side effects. After oral administration of these compounds, they can be biotransformed to stronger components and play a synergistic role with various chemotherapy drugs.

Over the last decades, traditional herbal medicines have been widely utilized for modern drug development. More and more studies have indicated that a daily intake of these herbal products could improve the life quality of patients (6, 7). Notably, a growing body of research suggests that traditional Chinese herbal can be regarded as effective adjuvant chemotherapy agents for improving the efficacy of cancer chemotherapy. In this review, ginseng, quercetin, and tea are the main research objects. The reasons why we have focused on these herbs are described as follows. First, these herbs are the most widely used traditional herbal medicines both in the East and West, and their beneficial effects have been extensively advertised. Another reason that has led us to pick these phytochemicals is that they are common in dietary supplements and have been confirmed to improve the life quality of hosts. The last and most important reason is that their multiple pharmacological properties, such as anti-oxidant, anti-inflammatory, and anti-cancer properties, have been widely recognized. Based on the above findings, we reviewed a large body of literature and concluded that all these herbs could effectively improve the effects of CRC chemotherapy. It should be noted that the chemical and pharmacological properties of ginseng, quercetin, and tea are completely independent of each other. Therefore, to avoid confusion, we discussed their properties and functions in great detail separately, as shown in Figure 1.

## Chemotherapy of CRC

With improvements in CRC treatment, multiple chemotherapeutic agents have been used in routine clinical practice; the chemotherapeutic agents, mainly including 5-fluorouracil (5-FU), irinotecan, oxaliplatin, and capecitabine, can be used either alone or in combination with each other, (8). Among them, 5-FU has historically been considered the foundation of the therapy for CRC, which has been used in clinical treatment for more than 60 years (9). An increasing number of clinical trials have demonstrated that 5-FU administered alone or in combination with other chemotherapeutic agents can significantly improve the survival rate of patients with CRC (10, 11). The response rate of 5-FU administered alone is only approximately 10–15% (12). However, combining 5-FU with other chemotherapeutic agents can effectively enhance curative effects and has been regarded as the first routine clinical practice. For example, leucovorin, a folinic acid derivative, can enhance the therapeutic response rate to 37% by suppressing the activation of thymidylate synthase (13). However, like many other common chemotherapy drugs, 5-FU also has many side effects, mainly including leukopenia, nausea, vomiting, hematopoietic depression, bone marrow suppression, neurotoxicity, and cardiotoxicity (14). In particular, leukopenia has been reckoned major dose-limiting toxicity of 5-FU administration occurs in approximately 93% of patients (15). In recent years, with a deepening understanding of drug properties, 5-FU has also shown stronger anti-cancer efficacy in clinical combinations with new-generation chemotherapy drugs. In conclusion, although 5-FU is an essential agent for treating both advanced and early-stage patients with CRC, its side effects cannot be ignored. Therefore, it is necessary to overcome these therapeutic challenges.

Capecitabine, an oral 5-FU prodrug that has been used in treating CRC for 20 years, can be enzymatically transformed into 5-FU at colorectal tumor sites after oral administration (16). Moreover, it has been demonstrated that even along administration of capecitabine exerts stronger chemotherapy effects and lower incidence of side effects than combined administration of 5-FU and leucovorin (17). However, capecitabine also has deficiencies, in particular the significantly increased incidence of the hand-foot syndrome and hyperbilirubinemia (18). As the most commonly used chemotherapy drugs for various malignant diseases, platinum-based agents have also been used in CRC treatment. Oxaliplatin, a third-generation platinum anti-cancer agent, is also a novel first-line treatment for metastatic CRC. It can inhibit the growth of tumor cells by inducing the formation of platinum-DNA adducts and eliciting a DNA damage response (19). The typical side effects include hematologic toxicity, gastrointestinal symptoms, and peripheral neuropathy (20).

Irinotecan is approved as second-line therapy for treating advanced/metastatic CRC, especially for patients who do not respond to the first-line 5-FU therapy (21). Its active metabolite SN-38, a camptothecin-based agent, can promote DNA damage and tumor cell apoptosis by binding with topoisomerase I, an important mediator of DNA transcription (22). The most common side effects of irinotecan treatment include myelosuppression, delayed-type diarrhea, cholinergic syndrome, vomiting, constipation, and neutropenia (23, 24). As shown above, each chemotherapeutic agent has its own properties and side effects. Over the past decade, sequential combination therapy with multiple chemotherapeutics has been the most standard chemotherapeutic treatment for CRC management; this therapy can promote the synergy of different agents and improve chemotherapy resistance using different action mechanisms (8, 25). For instance, the combined administration of oxaliplatin and irinotecan can be used as a salvage therapy for patients failing to respond to single-agent 5-FU treatment and is a first-line sequential treatment option for advanced CRC (26, 27). Although chemotherapeutic therapies have greatly improved the outcomes of patients with CRC, serious side effects and drug resistance are still major clinical challenges. In recent years, more and more drugs, especially traditional Chinese medicines, have been used to alleviate various side effects and improve chemoresistance.

## Ginseng

Ginseng is one of the most common traditional herbal medicines, which has been discovered in both East (Asian ginseng) and West (American ginseng) (28, 29). With the recent developments in the extraction process, multiple active components have been isolated from ginseng, mainly including ginsenosides, ginseng polysaccharides, flavonoids, polysaccharides, and ginseng polypeptides (30). Since ancient times, ginseng has been found to possess multiple pharmacological effects and has been used to treat various diseases, such as inflammation, cancers, metabolic syndromes, and autoimmune diseases. The anti-cancer effect of ginseng has attracted increasing interest and attention in the fields of various cancers, including ovarian cancer, CRC, breast cancer, lung cancer, prostate cancer, and liver cancer (31–34). Many in *vitro* and in *vivo* studies have demonstrated that ginseng or its extracts could significantly decrease the incidence of CRC and inhibit tumor growth (35). For example, Rg3, one of the most abundant and active ginsenosides can effectively inhibit the proliferation of CRC cells by suppressing the activity of the C/EBPβ/NF-κB signaling pathway (36). Similarly, another study also reported that ginsenoside Rg3 could inhibit the proliferation, migration and invasion of CRC cells and promote the apoptosis of these tumor cells by downregulating the expression of lncRNA CCAT1 (37). Other chemical compounds extracted from ginseng, such as flavonoids and polysaccharides, have been confirmed to have anti-CRC effects (38, 39). Recent studies further proposed that ginseng and its various constituents could improve the status of patients with CRC by increasing the efficiency of chemotherapy drugs (40, 41).

### Asian Ginseng, American Ginseng, and Panax Notoginseng

Ginseng and its extracts have great potential as chemotherapy adjuvant agents due to their low toxicity and strong anti-cancer properties (42). In particular, ginseng or its active components can enhance the sensitivity of chemotherapy and reduce its side effects. For instance, Fishbein et al. proposed that Asian ginseng could improve the anti-cancer function of 5-FU on HCT-116 human CRC cells (43). In addition, Panax notoginseng root extract, a remedy anti-cancer medicine, can also improve the chemopreventive functions of 5-FU and irinotecan in experiments *in vitro* (SW480) (44). These results are consistent with previous studies that notoginseng can enhance tumor radiosensitivity to the cytotoxic effect of ionizing radiation (45). In addition, another study reported that Panax notoginseng could increase the anti-proliferative ability of 5-FU on HCT-116 cells and significantly decrease the dosage of 5-FU required by CRC administration (46). Moreover, Li et al. reported that American ginseng berry extract could enhance the chemopreventive effect of 5-FU during CRC treatment both *in vivo* and *in vitro* (SW480, HCT-116 and HT-29), possibly by increasing cell arrest at S and G2/M phases (47).

Nausea and vomiting may be the most common adverse events in cancer chemotherapy treatment. For patients with oxaliplatin-based regimens, the incidence of nausea and vomiting is more than 70% (48). Previous studies reported that Korean red ginseng total extract could effectively attenuate cisplatin-induced nausea and vomiting in a ferret model (49). Further studies proposed that the anti-emetic effect of ginseng or its extracts was achieved by the antagonism of the 5-HT 3A receptor (50, 51). In a recent clinical trial, scholars investigated the curative effect of ginseng on nausea and vomiting induced by oxaliplatin-based regimens during CRC treatment, and they found that the administration of ginseng combined with some traditional medicines was capable of suppressing nausea and vomiting (52). In a randomized clinical phase III trial, Kim et al. proposed that Korean red ginseng administration could alleviate cancer-related fatigue in CRC patients with chemotherapy (53). Cancer-related fatigue, a common side effect of cancer chemotherapy treatment is a subjective physical feeling and can interfere with the sleep, mood, concentration, work, and daily life quality of patients (54). In this trial, 219 patients with mFOLFOX-6 administration chemotherapy were included in the Korean red ginseng treatment group, and other 219 patients treated with placebos were included in the control group. After 16-week administration, the results showed that Korean red ginseng treatment effectively improved fatigue, inhibited deterioration of fatigue-related life quality, and reduced the stress of these CRC patients receiving chemotherapy.

### Ginsenosides

Panaxadiol (PD), a diol-type ginsenoside derived from Panax ginseng or Panax pseudoginseng can also enhance the anti-cancer effects of 5-FU on CRC (55). The results showed that the combined administration of 5-FU and PD significantly exerted stronger anti-proliferative and pro-apoptotic abilities in the HCT-116 human CRC cell line than treatment with 5-FU alone. These results are consistent with a previous clinical study (56). Moreover, another *in vitro* study (HCT-116 and SW480) showed that PD could also enhance the anti-cancer effects of irinotecan, which might be achieved *via* inducing tumor cell apoptosis (57). This synergistic administration can effectively reduce the dose of irinotecan and the rate of side effects, indicating that some natural products are beneficial for CRC chemoadjuvant treatment.

Ginsenoside Rg3, a tetracyclic triterpenoid saponin with strong anti-cancer properties can inhibit the proliferation, invasion and migration of various tumors (58). For instance, one study reported that Rg3 could block the progression of colon cancer and promote the apoptosis of HT-29 colon cells by inhibiting the stemness of cancer stem cells, reducing tumor angiogenesis, and upregulating the AMPK pathway (59). In recent studies, scholars further proposed that Rg3 administration could significantly enhance the anti-cancer function of 5-FU both *in vivo* and *in vitro* (SW620 and LOVO) (60). After treatment with Rg3 and 5-FU together, this synergistic therapy was found to effectively suppress the proliferation, development and metastasis of tumors by activating the PI3K/Akt signaling pathway.

Protopanaxadiol (PPD), a secondary ginsenoside induced by a gut microbiome, can be bio-transformed by intestinal flora from ginseng extracts such as Rb1 and compound K (61, 62). According to a recent study, in addition to being able to inhibit tumor development directly, PPD can effectively enhance the effects of 5-FU on patients with CRC (62). It was found that the co-administration of PPD and 5-FU exerted stronger anti-proliferative and pro-apoptotic effects on HCT-116 human CRC cells than PPD or 5-FU alone treatment. A further *in vivo* experiment also confirmed that this co-administration could markedly reduce the tumor size in a dose-related manner.

## Quercetin

Quercetin (3, 3′, 4′, 5, 7-pentahydroxyflavone), a well-studied flavonoid in various vegetables and fruits is easily dissolved in the glacial acetic acid and aqueous solution (63). Hydrophilic glycoside, one of the most common constituents of quercetin extracts, cannot be directly absorbed by the host body and has to be transformed into quercetin metabolites by interacting with intestinal flora and key enzymes in digestive systems (64). Multiple pharmacological effects, including anti-inflammatory, anti-oxidative, anti-atherosclerosis, and anti-cancer effects, have been discovered in quercetin or its extracts (65). Further study demonstrated that quercetin could exert anti-cancer effects through various mechanisms, including inhibiting the activity of tyrosine kinase, regulating pathways involved in tumorigenesis, and interacting with specific proteins or receptors (66). It was found that quercetin and its derivatives could effectively inhibit tumor initiation and progression in both *in vivo* and *in vitro* CRC models (67). The molecular mechanisms are very complex and incompletely understood. According to previous studies, multiple signaling pathways were involved in the anti-cancer processes, such as Wnt/β-catenin, MAPK/JNK, NF-κB, and other related pathways (67). For instance, quercetin was reported to suppress the growth of multiple CRC cell lines (such as HT-29, Caco-2, DLD-1, and HCT-15) by blocking the activity of the AKT pathway (68–70). Another study further indicated that 3, 4-dihydroxyphenylacetic acid, a major derivative of quercetin, was capable of exerting CRC protective effects by reducing reactive oxygen species responses (71). In addition, recent studies reported that quercetin could be used as an effectively adjuvant chemotherapy agent for various cancer administration (65, 72–74). Especially in CRC chemotherapy, the synergistic effect of polyphenols has achieved relatively good potentiating effects.

A study in 1994 first reported that the combined administration of quercetin and 5-FU could significantly inhibit the growth of CRC cell line COLO 320DM cells (75). Recent studies further indicated that quercetin could increase the bioavailability of drugs by regulating the expression of key proteins associated with the development of drug resistance (76). Based on the above findings, Atashpour et al. proposed that quercetin treatment could enhance the cytotoxicity and apoptosis induction of doxorubicin in CRC stem cells and HT-29 cells by arresting tumor cells at the G2/M phase (77). Moreover, Han et al. reported that quercetin pretreatment could significantly promote the apoptosis of HT-29 cells induced by cisplatin, thus improving the anti-cancer functions of cisplatin during CRC administration (78). Further studies found that the combination of quercetin and cisplatin could directly activate the NF-κB signaling pathway to suppress cell proliferation and induce apoptosis (78). A recent study suggested that the combination of quercetin and luteolin, a member of the flavone group of flavonoids, could effectively increase the anti-cancer functions of 5-FU in HT-29 cells (79). Compared with the control group, this combination exerted stronger anti-proliferative and pro-apoptotic effects. This phenomenon was caused by suppressing angiogenesis and vasculogenesis. This combination modulated the apoptotic pathways and minimized the toxic effects of 5-FU.

P-glycoprotein-mediated multidrug resistance has been considered one of the most fundamental factors of cancer chemotherapy. Quercetin has been regarded as an inhibitor of P-glycoprotein-mediated multidrug resistance, which can overcome CRC resistance to chemotherapy *via* molecular mechanisms. For example, Zhou et al. reported that quercetin could effectively increase the cytotoxicity of doxorubicin to P-glycoprotein-overexpressed SW620/Ad300 cells by blocking D-glutamate metabolism and reducing the solute carrier family 1 member 5 (80). The CRC with microsatellite instability is resistant to 5-FU administration, which remains a clinical challenge. Xavier et al. first reported that quercetin treatment could effectively enhance the sensitivity of 5-FU on CO-115 and HCT-15 cells (81). After treatment with quercetin and 5-FU together, they found that the ratio of apoptotic cells significantly increased, which might be caused by special activation of the mitochondrial pathway.

Quercetin has been recognized as the most representative drug of flavonoids. In addition to quercetin, other flavonoids, such as epigallocatechin-3 gallate and isoflavone genistein, also possess chemopreventive properties (82, 83). Howells et al. further investigated whether the chemical modification of flavonol structures could enhance the pharmacological and toxicological properties of other flavonoids. They hypothesized that a flavonol molecule had no hydroxyl group on the A ring and only methoxyl groups on the B ring, which might possess cancer chemopreventive efficacy (84). To test this hypothesis, they produced a new compound, 3′, 4′, 5′-trimethoxyflavonol, a quercetin analog. Then, they compared the preclinical cancer chemopreventive properties of the new compound with those of two naturally flavonol congeners, quercetin and fisetin, *in vivo* (human-derived HCT-116 adenocarcinoma-bearing nude mice) and *in vitro* (APC10.1 cells derived from adenomas of *Apc*^*Min*^ mice). The result showed that the synthesized 3′, 4′, 5′-trimethoxyflavonol could significantly inhibit tumor proliferation and promote apoptosis by increasing wild-type p53 expression in two mouse models. The above studies also demonstrated that chemical modification might be an effective way to generate safe and efficacious cancer chemopreventive agents.

Moreover, one study reported that quercetin treatment could also effectively enhance the radio sensitivity of CRC in addition to improving the chemotherapy sensitivity. They found that the pretreatment of quercetin enabled colorectal cells to be more sensitive to radiotherapy by downregulating the ataxia–telangiectasia-mutated-related signaling pathways and promoting irradiation-induced γ-H2AX and 53BP1 focus formation (85). A recent study indicated that the combination of quercetin and ionizing radiation could have greater therapeutic potential for CRC, which is consistent with the above results (86). The detailed mechanism included directly suppressing the Notch-1 signaling pathway and targeting colon cancer stem cells, one group of rare immortal cells involved in radiation therapy resistance.

## Tea

Tea is a commonly consumed beverage derived from the leaves and leaf buds of the Camellia sinensis. Tea has been studied extensively in health and disease fields, such as preventing hypertension and cardiovascular diseases, reducing obesity, treating metabolic syndromes, mediating gut microbiotas, and preventing and treating cancers (87). There are many types of tea, such as black tea, green tea, Pu-erh tea, white tea, yellow tea, oolong tea, and dark tea, all of which were produced *via* different methods (88). For instance, green tea, also named non-fermented tea is produced from dried green tea leaves. Black tea also named most or fully fermented tea is obtained from extensively solid-state fermentation involving microorganisms. The partially fermented tea is named oolong tea (89). In the last few years, various chemical components, mainly including catechin derivatives, polysaccharides, pigments, theophylline, glycosides, phenolic acids, and alkaloids have been isolated from tea. Catechins, such as (–)-epigallocatechin-3-gallate (EGCG), (–)-epigallocatechin (EGC), (–)-epicatechin-3-gallate (ECG), and (–)-epicatechin, have been studied extensively in cancer prevention and administration. Previous studies reported that tea and its components could exert anti-cancer effects through various signaling and metabolic mechanisms, such as inhibiting tumorigenesis, promoting apoptosis, regulating proliferation transformation, and targeting key transmembrane receptors or kinases (87). It has been confirmed that EGCG could inhibit CRC initiation and progression by reducing oxidative reaction and promoting tumor cell apoptosis (90). Some meta-analyses also showed that tea consumption was closely associated with CRC risk (91, 92). Based on these results, some scholars further proposed that tea and tea polyphenols could be used as promising chemopreventive agents for CRC treatment (93).

### EGCG and EGC

(–)-epigallocatechin-3-gallate is a major green tea polyphenol and is regarded as an important tumor inhibitor in various cancers (94). It has been shown that the combinations of EGCG and other catechins can exert relatively strong anti-cancer effects in both *in vitro* and *in vivo* experiments (95). Some studies indicated that EGCG could improve chemoresistance and reduce tumor recurrence. For instance, Toden et al. found that EGCG treatment could sensitize chemoresistant CRC cells (HCT-116 and SW480) to standard 5-FU administration. Specific chemopreventive activities include increasing 5-FU-induced cytotoxicity and suppressing the growth of tumor cells by triggering apoptosis and promoting cell cycle arrest (96). La et al. reported that EGCG administration could effectively increase the chemosensitivity of 5-FU in HCT-116 and DLD1 cell lines by suppressing tumor growth, promoting apoptosis, and causing DNA damage, which is consistent with the above results (97). According to further mechanistic studies, EGCG can upregulate the expression of NF-κB and miR-155-5p by blocking GRP78 activity, further suppressing the protein expression of MDR1 and increasing the 5-FU accumulation in CRC cells. In another study, Shimizu et al. proposed that EGCG could exert chemopreventive effects by inhibiting the activity of signaling pathways related to receptor tyrosine kinases, such as EGFR, IGF-1R, and VEGFR2 signaling pathways (98). Moreover, combining EGCG with cisplatin or oxaliplatin could significantly inhibit the proliferation of DLD-1 and HT-29 cells and reduce cytotoxic effects by regulating autophagy-related signaling pathways (93).

Irinotecan is a common DNA-damaging chemotherapeutic agent for CRC treatment, the use of which is limited by its low solubility and high toxicity. Combined with the previous studies, they found that the co-administration of EGCG and Gefitinib or Bleomycin could reduce their dose and resistance (99, 100). Wu et al. further investigated the synergy of EGCG and irinotecan on CRC treatment (101). They treated CRC cells RKO and HCT116 with EGCG and irinotecan together, and the results showed that the combined administration exerted relatively strong inhibitory effects on the proliferation, migration, and invasion of tumor cells. The specific molecular mechanism includes inducing S- or G2-phase arrest and causing more extensive DNA damage. Moreover, a study reported that EGCG and EGC could increase the chemosensitivity of low-dose doxorubicin both *in vivo* and *in vitro* (SW620) by blocking the activation of protein kinase C, a drug resistance-related protein (102). According to some studies, in addition to directly improving chemotherapy responses, tea nanoparticles can be used to deliver chemotherapeutic agents for cancer treatment. For instance, Wang et al. proposed that tea nanoparticles, a safe nanocarrier with good biocompatibility and low toxicity could load doxorubicin into tumors, thus enhancing its intertumoral accumulation and improving its chemotherapy efficacy in an animal study (103).

### Chemopreventive Effects

The view that drinking tea can prevent cancer has been proposed for many years. For instance, Shimizu et al. reported that consuming proper green tea every day could inhibit the recurrence of CRC (104). Another study showed that green tea catechins could prevent CRC through multiple molecular mechanisms, including decreasing detergent-insoluble membrane domain, inhibiting the activity of the specific receptor tyrosine kinases (such as EGFR, IGF-1R, and VEGFR-2), and reducing the expression of hypoxia-inducible factor 1a (HIF1a), IGF-1, IGF2, and EGF (98, 105). In a randomized controlled trial, Henning et al. proposed that tea polyphenols could be transformed into phenolic metabolites by the colonic microflora, thus playing a significant role in CRC prevention (106). Ku-jin tea, a very popular beverage in the world, is an essential anti-inflammatory and anti-oxidative regulator and can also play chemopreventive effects on CRC. In a CRC rat model induced by azoxymethane, Bi et al. found that long-term treatment with Ku-jin tea could significantly decrease the number of aberrant crypts, aberrant crypt foci (ACF), and crypts/foci in rats through regulating metabolism-associated pathways, further indicating that Ku-jin tea can be used as a promising chemopreventive agent for CRC chemoprevention (107).

## Perspectives and Conclusions

The synergistic therapy of herbal medicines combined with chemotherapy may revolutionize cancer treatment. With the development of precision medicine, chemotherapy has played an increasingly important role in clinical cancer treatment (108, 109). Especially for CRC, various chemotherapeutic regimens have been proposed and have achieved remarkable clinical efficacy. For example, for lymph node-positive patients, the FOLFOX regimen (5-FU, leucovorin, and oxaliplatin) is recommended (110). In terms of locally advanced rectal cancer, neoadjuvant chemoradiation therapy with 5-FU and radiation therapy should be considered for the patients. For patients with metastatic CRC, FOLFOX, or FOLFIRI (5-FU, leucovorin, and irinotecan) regimens are recommended as standard first-line treatment choices (111).

Traditional herbal medicines, exerting huge therapeutic potential in various diseases, are promising adjuvant chemotherapy agents. In this review, to allow readers to quickly know this field, we selected the three most studied herbs (ginseng, quercetin, and tea) as representative drugs to conclude their synergies in CRC chemotherapy administration (Table 1). By summing up the points, we discovered that most studies were focused on investigating the synergistic effects of the three herbs on 5-FU, the most commonly used chemotherapy drug for CRC treatment. As expected, we found that all the three herbs and their major extracts could significantly enhance the chemopreventive functions of 5-Fu and reduce its side effects. In terms of ginseng, there are three most common species, including Asian ginseng, American ginseng, and Panax notoginseng, all of which have been confirmed to possess synergistic effects. Their major components, such as ginsenoside Rg3, PPT, and PPD, also possess stronger pharmacological and biological effects. In addition, the synergistic effects of them and other chemotherapy drugs, such as irinotecan, have also been studied both *in vivo* and *in vitro*. Quercitrin is commonly found in plant foods used to treat various diseases, especially, which has been identified to be an effectively antitumor agent. Herein, we have summarized that quercetin treatment could enhance the cytotoxicity of 5-FU, cisplatin, and doxorubicin by regulating different molecular mechanisms. The research on tea was mainly focused on EGCG, a major tea polyphenol, which can improve the chemotherapy efficacy of 5-FU, irinotecan, cisplatin, and oxaliplatin and play chemopreventive effects both *in vivo* and *in vitro*. However, its detailed mechanisms, mainly including promoting proliferation and inhibiting apoptosis by regulating related signaling pathways, are not fully understood.

**Table 1:** The synergistic effects of ginseng, quercetin, and tea on chemotherapy treatment of colorectal cancer.

**Herbs or their composition**	**Studied objects**	**Chemotherapeutics**	**Effects**	**Refs**.
Asian ginseng	HCT-116	5-fluorouracil	Improving efficacy of chemotherapy.	(43)
Panax notoginseng root extract	SW-480	5-fluorouracil Irinotecan	Improving efficacy of chemotherapy.	(44)
Panax notoginseng	HCT-116	5-fluorouracil	Improving efficacy of chemotherapy.	(46)
American ginseng berry extract	SW-480, HCT-116 HT-29, Animal model	5-fluorouracil	Improving efficacy of chemotherapy.	(47)
Korean red ginseng	Animal model	Cisplatin	Reducing side effects of chemotherapy.	(49)
Asian ginseng	Clinical trial	Oxaliplatin	Reducing side effects of chemotherapy.	(52).
Korean red ginseng	Clinical trial	mFOLFOX-6	Reducing side effects of chemotherapy.	(53)
Panaxadiol	HCT-116	5-fluorouracil	Improving efficacy of chemotherapy.	(55)
Panaxadiol	HCT-116, SW-480	Irinotecan	Improving efficacy of chemotherapy.	(57)
Ginsenoside Rg3	SW620, LOVO, Animal model	5-fluorouracil	Improving efficacy of chemotherapy.	(60)
Protopanaxadiol	HCT-116	5-fluorouracil	Improving efficacy of chemotherapy.	(62)
Quercetin	COLO 320 DM	5-fluorouracil	Improving efficacy of chemotherapy.	(75)
Quercetin	HT-29	Doxorubicin	Improving efficacy of chemotherapy.	(77)
Quercetin	HT-29	Cisplatin	Improving efficacy of chemotherapy.	(78)
Quercetin	HT-29	5-fluorouracil	Improving efficacy of chemotherapy.	(79)
Quercetin	SW620, Ad300	Doxorubicin	Increases sensitivity to chemotherapy	(80)
Quercetin	CO-115, HCT-15	5-fluorouracil	Increases sensitivity to chemotherapy	(81)
(–)-epigallocatechin-3-gallate	HCT116, SW480	5-fluorouracil	Increases sensitivity to chemotherapy	(96)
(–)-epigallocatechin-3-gallate	HCT-116, DLD1	5-fluorouracil	Increases sensitivity to chemotherapy	(97)
(–)-epigallocatechin-3-gallate	DLD-1, HT-29	Cisplatin Oxaliplatin	Reducing side effects of chemotherapy.	(93)
(–)-epigallocatechin-3-gallate	RKO and HCT-116	Irinotecan	Improving efficacy of chemotherapy.	(101)
(–)-epigallocatechin-3-gallate	SW620	Doxorubicin	Increases sensitivity to chemotherapy	(102)
Tea nanoparticle	Animal model	Doxorubicin	Improving efficacy of chemotherapy.	(103)

Although many preclinical studies have been performed, the clinical applications of these herbs are still limited due to too many unknown variables. In the future, an increasing number of studies should be performed to clarify specific mechanisms and develop more effective chemopreventive agents for CRC administration.

## Author Contributions

LZ drafted the review. HZ and MS generated the graphs. NL guided the construction of the manuscript. YZ edited the review. KZ, JL, and PL provided input on the scope and content of the review. All the authors contributed to the article and approved the submitted version.

## Conflict of Interest

The authors declare that the research was conducted in the absence of any commercial or financial relationships that could be construed as a potential conflict of interest.

## Publisher's Note

All claims expressed in this article are solely those of the authors and do not necessarily represent those of their affiliated organizations, or those of the publisher, the editors and the reviewers. Any product that may be evaluated in this article, or claim that may be made by its manufacturer, is not guaranteed or endorsed by the publisher.

## Abbreviations

CRC: colorectal cancer
5-FU: 5-fluorouracil
PD: panaxadiol
PPD: protopanaxadiol
EGCG: (–)-epigallocatechin-3-gallate
EGC: (–)-epigallocatechin
ECG: (–)-epicatechin-3-gallate.
